# Hexa-μ_2_-acetato-κ^12^ *O*:*O*′-(azido-κ*N*)bis­(methanol-κ*O*)-μ_3_-oxido-trichromium(III) methanol monosolvate

**DOI:** 10.1107/S1600536811039699

**Published:** 2011-10-05

**Authors:** Guo-Qing Jiang, Jian-Hua Li, Miao Wang, Yu-Jun Shi

**Affiliations:** a College of Chemistry and Chemical Engineering, Nantong University, Nantong 226019, People’s Republic of China

## Abstract

In the crystal structure of the title complex, [Cr_3_(CH_3_CO_2_)_6_(N_3_)O(CH_3_OH)_2_]·CH_3_OH, the trinuclear core has a central O atom; two methanol mol­ecules and an azide ion are coordinated to the Cr^III^ atoms in the core. The three Cr^III^ atoms form vertices of a nearly equilateral triangle. Each of the six acetate carboxyl­ate groups bridges a Cr—O—Cr fragment. In the crystal, the molecules inter­act with methanol solvent mol­ecules through O—H⋯O and O—H⋯N hydrogen bonds, forming a two-dimensional hydrogen-bonded network parallel to (100).

## Related literature

The design and synthesis of bioactive organic metal complexes have attracted attention since the deficiency of certain trace metals can cause diseases and disorders, see: Farrel (1999 ▶). For the supplementation of animal diets with chromium, see: Vincent (2000 ▶). For chromium carboxyl­ate complexes, see: Anson *et al.* (1997 ▶); Chang & Jeffrey (1970 ▶); Fujihara *et al.* (1998 ▶). For the synthesis of the starting material, [Cr_3_O(CH_3_CO_2_)_6_(H_2_O)_3_]Cl·6H_2_O, see: Earnshaw *et al.* (1966 ▶).
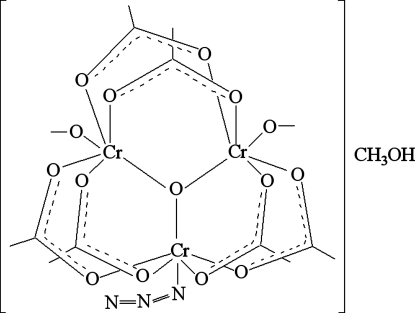


## Experimental

### 

#### Crystal data

[Cr_3_(C_2_H_3_O_2_)_6_(N_3_)O(CH_4_O)_2_]·CH_4_O
*M* *_r_* = 664.42
Monoclinic, 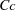

*a* = 21.165 (5) Å
*b* = 10.609 (3) Å
*c* = 15.641 (4) Å
β = 130.484 (4)°
*V* = 2671.2 (12) Å^3^
*Z* = 4
Mo *K*α radiation
μ = 1.27 mm^−1^
*T* = 153 K
0.20 × 0.16 × 0.12 mm

#### Data collection

Bruker SMART APEX CCD area-detector diffractometer
Absorption correction: multi-scan (*SADABS*; Bruker, 2000 ▶) *T*_min_ = 0.776, *T*_max_ = 0.859
6800 measured reflections
3692 independent reflections
3155 reflections with *I* > 2σ(*I*)
*R*_int_ = 0.034

#### Refinement

*R*[*F* ^2^ > 2σ(*F* ^2^)] = 0.059
*wR*(*F* ^2^) = 0.130
*S* = 1.08
3692 reflections
343 parameters
986 restraints
H-atom parameters constrained
Δρ_max_ = 0.49 e Å^−3^
Δρ_min_ = −0.47 e Å^−3^
Absolute structure: Flack (1983 ▶), 1110 Friedel pairs
Flack parameter: 0.05 (3)

### 

Data collection: *SMART* (Bruker, 2000 ▶); cell refinement: *SAINT* (Bruker, 2000 ▶); data reduction: *SAINT*; program(s) used to solve structure: *SHELXS97* (Sheldrick, 2008 ▶); program(s) used to refine structure: *SHELXL97* (Sheldrick, 2008 ▶); molecular graphics: *SHELXTL* (Sheldrick, 2008 ▶); software used to prepare material for publication: *SHELXTL*.

## Supplementary Material

Crystal structure: contains datablock(s) global, I. DOI: 10.1107/S1600536811039699/ds2137sup1.cif

Structure factors: contains datablock(s) I. DOI: 10.1107/S1600536811039699/ds2137Isup2.hkl

Additional supplementary materials:  crystallographic information; 3D view; checkCIF report

## Figures and Tables

**Table 1:** Hydrogen-bond geometry (Å, °)

*D*—H⋯*A*	*D*—H	H⋯*A*	*D*⋯*A*	*D*—H⋯*A*
O16—H16*F*⋯N1^i^	0.96	1.85	2.734 (4)	151
O16—H16*F*⋯O3^i^	0.96	2.36	2.972 (3)	122
O16—H16*F*⋯O9^i^	0.96	2.54	3.254 (5)	132
O16—H16*F*⋯N2^i^	0.96	2.63	3.370 (5)	134
O15—H15*A*⋯N1^ii^	0.83	1.91	2.621 (5)	144
O15—H15*A*⋯N2^ii^	0.83	2.58	3.332 (5)	152
O14—H14*D*⋯O16	0.82	2.03	2.602 (5)	126

Symmetry codes: (i) 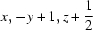; (ii) 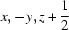.

**Fig. 1.**
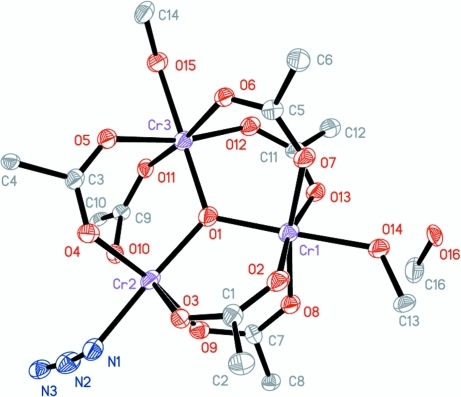
View of the title compound, with the atomic numbering scheme. Displacement ellipsoids are drawn at the 50% probability level.

**Fig. 2.**
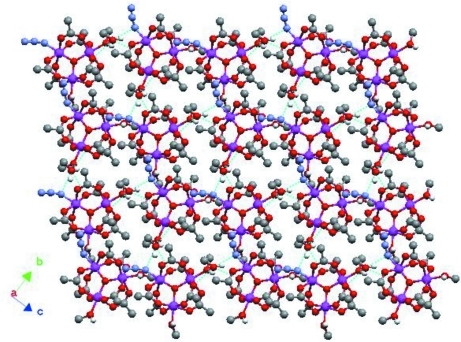
View of the 2D H bonded network of (I) along [100], showing molecules connected by O—H···O and O—H ···N hydrogen bonds (dashed lines). H atoms not involved in hydrogen bonding have been omitted.

## supplementary crystallographic information

### Comment

The design and synthesis of bioactive organic metal complexes have attracted intense attention in recent years, since the deficiency of certain trace minerals can cause different diseases and disorders (Farrel, 1999). Among these, Chromium (III) is known to activate enzymes, maintain protein stability and enhance carbohydrate metabolism. Nutritionists believe organic chromium should be supplemented in most animal diets (Vincent, 2000). In the past, chromium carboxylate complexes, in particular the acetate complexes have been extensively studied and characterized (Chang & Jeffrey, 1970; Anson *et al.*, 1997; Fujihara *et al.*, 1998). In order to achieve transition metal complexes by self-assembly, and to explore the relationship between the structure and the biological properties, as one part of our systematic work, in this paper, we report on the synthesis and crystal structure of the title compound, (I)(Fig. 1).

In (I), all the Cr(III) ions have octahedral environment; the equatorial positions in two of the metal atoms are occupied by the carboxylate O atoms and the apical positions are coordinated by the O atoms of MeOH and center O atom. In the other Cr(III) atom, one of the axial positions is occupied by the N atom of the azide ion. In the crystal structure, intermolecular O—H···O and O—H ···N hydrogen bonds link the molecules into two-dimensional network. (Table 1, Figure 2)

### Experimental

The starting material [Cr_3_O (C_2_H_3_O_2_)_6_(H_2_O)_3_] Cl.6H_2_O was prepared by using a literature method (Earnshaw *et al.*, 1966). [Cr_3_O (C_2_H_3_O_2_)_6_(H_2_O)_3_]Cl^.^6H_2_O (0.8 g, 1.0 mmol) was dissolved in methanol (10 ml) and then NaN_3_ (0.13 g, 2 mmol) was added. After refluxing for 2 h, the solution was filtered and the dark-green crystals were obtained by the solvent molecules allowed to evaporate slowly.

### Refinement

Carbon-bound H atoms were positioned geometrically, with C—H = 0.97Å for methylene and 0.93 Å for aromatic, and refined using a riding model, with *U*_iso_ (H) = 1.2 *U*_eq_ (C). The hydroxyl H atom was positioned geometrically and freely refined.

### Crystal data

**Table:**

[Cr_3_(C_2_H_3_O_2_)_6_(N_3_)O(CH_4_O)_2_]·CH_4_O	*Z* = 4
*M**_r_* = 664.42	*F*(000) = 1364
Monoclinic, *C**c*	*D*_x_ = 1.652 Mg m^−^^3^
Hall symbol: C -2yc	Mo *K*α radiation, λ = 0.71073 Å
*a* = 21.165 (5) Å	θ = 2.3–28.5°
*b* = 10.609 (3) Å	µ = 1.27 mm^−^^1^
*c* = 15.641 (4) Å	*T* = 153 K
β = 130.484 (4)°	Prism, green
*V* = 2671.2 (12) Å^3^	0.2 × 0.16 × 0.12 mm

### Data collection

**Table:**

Bruker SMART APEX CCD area-detector diffractometer	3692 independent reflections
Radiation source: fine-focus sealed tube	3155 reflections with *I* > 2σ(*I*)
graphite	*R*_int_ = 0.034
φ and ω scans	θ_max_ = 25.9°, θ_min_ = 2.3°
Absorption correction: multi-scan (*SADABS*; Bruker, 2000)	*h* = −23→23
*T*_min_ = 0.776, *T*_max_ = 0.859	*k* = −12→13
6800 measured reflections	*l* = −18→19

### Refinement

**Table:**

Refinement on *F*^2^	Secondary atom site location: difference Fourier map
Least-squares matrix: full	Hydrogen site location: inferred from neighbouring sites
*R*[*F*^2^ > 2σ(*F*^2^)] = 0.059	H-atom parameters constrained
*wR*(*F*^2^) = 0.130	*w* = 1/[σ^2^(*F*_o_^2^) + (0.0594*P*)^2^ + 7.9006*P*] where *P* = (*F*_o_^2^ + 2*F*_c_^2^)/3
*S* = 1.08	(Δ/σ)_max_ < 0.001
3692 reflections	Δρ_max_ = 0.49 e Å^−^^3^
343 parameters	Δρ_min_ = −0.47 e Å^−^^3^
986 restraints	Absolute structure: Flack (1983), 1110 Friedel pairs
Primary atom site location: structure-invariant direct methods	Flack parameter: 0.05 (3)

### Special details

**Table:**

Geometry. All esds (except the esd in the dihedral angle between two l.s. planes) are estimated using the full covariance matrix. The cell esds are taken into account individually in the estimation of esds in distances, angles and torsion angles; correlations between esds in cell parameters are only used when they are defined by crystal symmetry. An approximate (isotropic) treatment of cell esds is used for estimating esds involving l.s. planes.
Refinement. Refinement of F^2^ against ALL reflections. The weighted R-factor wR and goodness of fit S are based on F^2^, conventional R-factors R are based on F, with F set to zero for negative F^2^. The threshold expression of F^2^ > 2sigma(F^2^) is used only for calculating R-factors(gt) etc. and is not relevant to the choice of reflections for refinement. R-factors based on F^2^ are statistically about twice as large as those based on F, and R- factors based on ALL data will be even larger.

### Fractional atomic coordinates and isotropic or equivalent isotropic displacement parameters (Å^2^)

**Table:**

	*x*	*y*	*z*	*U*_iso_*/*U*_eq_	
C1	0.30660 (19)	0.3957 (4)	0.2755 (3)	0.0348 (7)	
C2	0.3497 (2)	0.4770 (4)	0.2475 (3)	0.0456 (11)	
H2A	0.3162	0.5498	0.2063	0.068*	
H2B	0.3574	0.4295	0.2027	0.068*	
H2C	0.4027	0.5033	0.3158	0.068*	
C3	0.31584 (19)	−0.0377 (3)	0.3313 (3)	0.0333 (10)	
C4	0.36487 (16)	−0.1389 (3)	0.3277 (2)	0.0270 (8)	
H4A	0.3272	−0.1958	0.2668	0.041*	
H4B	0.3983	−0.1847	0.3974	0.041*	
H4C	0.4001	−0.1002	0.3167	0.041*	
C5	0.39185 (18)	0.2443 (4)	0.6047 (3)	0.0320 (9)	
C6	0.4793 (2)	0.2640 (4)	0.7168 (3)	0.0409 (11)	
H6A	0.5120	0.3096	0.7045	0.061*	
H6B	0.5046	0.1836	0.7499	0.061*	
H6C	0.4764	0.3111	0.7665	0.061*	
C7	0.09410 (16)	0.3896 (3)	0.1609 (2)	0.0290 (8)	
C8	0.01608 (18)	0.4630 (3)	0.0702 (3)	0.0315 (9)	
H8A	−0.0152	0.4787	0.0939	0.047*	
H8B	−0.0171	0.4149	0.0019	0.047*	
H8C	0.0311	0.5417	0.0575	0.047*	
C9	0.10500 (15)	−0.0215 (3)	0.2246 (2)	0.0254 (7)	
C10	0.02352 (17)	−0.0967 (3)	0.1558 (3)	0.0336 (9)	
H10A	0.0228	−0.1487	0.2053	0.050*	
H10B	0.0195	−0.1488	0.1023	0.050*	
H10C	−0.0226	−0.0394	0.1168	0.050*	
C11	0.1791 (2)	0.2515 (4)	0.4951 (3)	0.0357 (10)	
C12	0.1373 (2)	0.2719 (4)	0.5457 (3)	0.0425 (11)	
H12A	0.1577	0.3485	0.5888	0.064*	
H12B	0.1499	0.2022	0.5938	0.064*	
H12C	0.0783	0.2778	0.4863	0.064*	
C13	0.2572 (2)	0.6577 (4)	0.3982 (3)	0.0376 (11)	
H13A	0.2727	0.6325	0.3552	0.056*	
H13B	0.2969	0.7173	0.4546	0.056*	
H13C	0.2031	0.6958	0.3494	0.056*	
C14	0.3548 (2)	−0.1309 (4)	0.6294 (3)	0.0441 (12)	
H14A	0.3815	−0.1580	0.6010	0.066*	
H14B	0.3481	−0.2016	0.6612	0.066*	
H14C	0.3883	−0.0679	0.6862	0.066*	
C16	0.0674 (3)	0.6046 (5)	0.3771 (4)	0.0615 (15)	
H16A	0.0340	0.6648	0.3781	0.092*	
H16B	0.0473	0.5211	0.3709	0.092*	
H16C	0.0641	0.6211	0.3139	0.092*	
Cr1	0.24501 (3)	0.37094 (6)	0.39698 (4)	0.03574 (16)	
Cr2	0.19595 (3)	0.16932 (5)	0.20163 (5)	0.03655 (17)	
Cr3	0.25541 (3)	0.06601 (6)	0.44353 (4)	0.03542 (16)	
N1	0.16124 (17)	0.1451 (3)	0.0481 (3)	0.0411 (10)	
N2	0.09146 (19)	0.0877 (4)	−0.0301 (3)	0.0486 (11)	
N3	0.04438 (17)	0.0238 (3)	−0.0905 (3)	0.0415 (9)	
O1	0.23315 (13)	0.2032 (2)	0.3494 (2)	0.0366 (6)	
O2	0.30800 (13)	0.4304 (2)	0.3528 (2)	0.0381 (6)	
O3	0.26887 (12)	0.2994 (2)	0.21374 (18)	0.0318 (5)	
O4	0.28633 (13)	0.0431 (2)	0.25849 (19)	0.0351 (6)	
O5	0.31318 (13)	−0.0395 (2)	0.40820 (19)	0.0341 (6)	
O6	0.36588 (13)	0.1342 (2)	0.57644 (19)	0.0341 (6)	
O7	0.35263 (15)	0.3426 (2)	0.5511 (2)	0.0414 (7)	
O8	0.14220 (14)	0.4327 (2)	0.2559 (2)	0.0364 (7)	
O9	0.10370 (13)	0.2883 (2)	0.12721 (19)	0.0331 (6)	
O10	0.12087 (14)	0.0348 (3)	0.16931 (19)	0.0378 (6)	
O11	0.14884 (12)	−0.0185 (2)	0.32863 (18)	0.0324 (6)	
O12	0.20495 (13)	0.1442 (2)	0.5015 (2)	0.0378 (7)	
O13	0.18466 (13)	0.3474 (2)	0.4524 (2)	0.0364 (6)	
O14	0.25539 (16)	0.5504 (2)	0.4508 (2)	0.0397 (7)	
O15	0.27862 (14)	−0.0813 (3)	0.5434 (2)	0.0417 (7)	
O16	0.14976 (14)	0.6145 (3)	0.4761 (2)	0.0399 (8)	
H16F	0.1548	0.6836	0.5198	0.060*	
H14D	0.2535	0.5694	0.4998	0.060*	
H15A	0.2419	−0.0669	0.5470	0.060*	

### Atomic displacement parameters (Å^2^)

**Table:**

	*U*^11^	*U*^22^	*U*^33^	*U*^12^	*U*^13^	*U*^23^
C1	0.0278 (9)	0.0326 (15)	0.0316 (7)	0.0037 (10)	0.0137 (6)	0.0048 (9)
C2	0.0432 (12)	0.0458 (19)	0.0370 (13)	−0.0092 (13)	0.0213 (9)	−0.0077 (13)
C3	0.0290 (11)	0.0250 (14)	0.0300 (12)	0.0024 (11)	0.0121 (8)	0.0045 (11)
C4	0.0308 (9)	0.0293 (15)	0.0256 (10)	−0.0053 (10)	0.0204 (7)	−0.0066 (11)
C5	0.0272 (10)	0.0348 (16)	0.0326 (12)	0.0012 (12)	0.0188 (8)	−0.0020 (12)
C6	0.0338 (12)	0.0416 (18)	0.0363 (14)	−0.0018 (14)	0.0179 (10)	0.0014 (14)
C7	0.0255 (9)	0.0349 (16)	0.0303 (10)	0.0058 (11)	0.0198 (7)	0.0067 (11)
C8	0.0308 (11)	0.0261 (15)	0.0309 (12)	0.0075 (12)	0.0170 (8)	−0.0005 (11)
C9	0.0261 (8)	0.0277 (15)	0.0341 (8)	0.0003 (9)	0.0247 (6)	−0.0053 (9)
C10	0.0372 (10)	0.0294 (16)	0.0389 (12)	−0.0152 (11)	0.0268 (8)	−0.0164 (11)
C11	0.0319 (11)	0.0310 (15)	0.0283 (12)	0.0020 (12)	0.0125 (8)	−0.0036 (12)
C12	0.0353 (12)	0.0362 (18)	0.0385 (14)	0.0067 (14)	0.0161 (9)	−0.0006 (14)
C13	0.0359 (13)	0.0311 (17)	0.0316 (13)	−0.0065 (13)	0.0156 (10)	−0.0092 (13)
C14	0.0360 (13)	0.0302 (17)	0.0481 (16)	−0.0055 (13)	0.0192 (10)	0.0020 (14)
C16	0.0601 (18)	0.042 (2)	0.0531 (19)	0.0028 (18)	0.0239 (13)	−0.0114 (17)
Cr1	0.03062 (18)	0.0319 (3)	0.0343 (2)	−0.0015 (2)	0.01643 (14)	−0.0020 (2)
Cr2	0.0327 (2)	0.0252 (2)	0.0313 (2)	−0.0022 (2)	0.01160 (16)	−0.0014 (2)
Cr3	0.03091 (18)	0.0307 (3)	0.0328 (2)	−0.0003 (2)	0.01535 (15)	−0.0001 (2)
N1	0.0346 (12)	0.0321 (15)	0.0345 (12)	−0.0021 (12)	0.0125 (9)	0.0019 (12)
N2	0.0338 (12)	0.0470 (17)	0.0418 (14)	−0.0075 (13)	0.0142 (10)	−0.0084 (13)
N3	0.0349 (11)	0.0421 (16)	0.0385 (12)	−0.0112 (12)	0.0198 (8)	−0.0107 (12)
O1	0.0333 (7)	0.0309 (9)	0.0317 (6)	−0.0027 (8)	0.0149 (5)	0.0006 (7)
O2	0.0335 (6)	0.0320 (9)	0.0355 (6)	−0.0059 (8)	0.0165 (5)	−0.0047 (7)
O3	0.0332 (6)	0.0327 (9)	0.0284 (7)	−0.0041 (6)	0.0195 (5)	−0.0006 (7)
O4	0.0372 (7)	0.0300 (9)	0.0292 (7)	0.0077 (7)	0.0176 (6)	0.0013 (7)
O5	0.0329 (7)	0.0278 (9)	0.0303 (8)	0.0021 (7)	0.0155 (6)	0.0022 (7)
O6	0.0336 (7)	0.0285 (9)	0.0296 (8)	−0.0017 (8)	0.0158 (6)	−0.0012 (8)
O7	0.0365 (8)	0.0293 (9)	0.0323 (8)	−0.0001 (8)	0.0107 (6)	−0.0012 (8)
O8	0.0358 (8)	0.0286 (9)	0.0312 (8)	0.0040 (8)	0.0156 (6)	0.0009 (8)
O9	0.0314 (7)	0.0325 (9)	0.0280 (7)	0.0044 (7)	0.0160 (5)	−0.0011 (7)
O10	0.0378 (6)	0.0356 (8)	0.0287 (7)	−0.0088 (5)	0.0166 (5)	0.0026 (7)
O11	0.0288 (7)	0.0299 (9)	0.0337 (7)	−0.0056 (7)	0.0181 (5)	0.0023 (7)
O12	0.0316 (7)	0.0329 (10)	0.0367 (8)	−0.0005 (8)	0.0168 (6)	−0.0027 (8)
O13	0.0370 (7)	0.0281 (9)	0.0345 (8)	0.0016 (8)	0.0189 (6)	−0.0010 (7)
O14	0.0392 (7)	0.0316 (9)	0.0305 (8)	0.0013 (8)	0.0146 (6)	−0.0029 (8)
O15	0.0376 (8)	0.0313 (10)	0.0339 (9)	0.0031 (8)	0.0133 (6)	0.0023 (8)
O16	0.0378 (9)	0.0369 (12)	0.0277 (9)	−0.0048 (10)	0.0135 (7)	−0.0136 (9)

### Geometric parameters (Å, °)

**Table:**

C1—O2	1.245 (5)	C13—O14	1.419 (5)
C1—O3	1.270 (4)	C13—H13A	0.9600
C1—C2	1.511 (7)	C13—H13B	0.9600
C2—H2A	0.9600	C13—H13C	0.9600
C2—H2B	0.9600	C14—O15	1.367 (4)
C2—H2C	0.9600	C14—H14A	0.9600
C3—O4	1.224 (4)	C14—H14B	0.9600
C3—O5	1.241 (5)	C14—H14C	0.9600
C3—C4	1.519 (5)	C16—O16	1.394 (4)
C4—H4A	0.9600	C16—H16A	0.9600
C4—H4B	0.9600	C16—H16B	0.9600
C4—H4C	0.9600	C16—H16C	0.9600
C5—O6	1.243 (4)	Cr1—O1	1.881 (3)
C5—O7	1.256 (4)	Cr1—O8	1.949 (2)
C5—C6	1.527 (4)	Cr1—O2	1.961 (3)
C6—H6A	0.9600	Cr1—O13	1.970 (3)
C6—H6B	0.9600	Cr1—O7	1.990 (2)
C6—H6C	0.9600	Cr1—O14	2.035 (3)
C7—O8	1.220 (4)	Cr2—O1	1.931 (3)
C7—O9	1.270 (5)	Cr2—O10	1.943 (3)
C7—C8	1.517 (4)	Cr2—O9	1.952 (2)
C8—H8A	0.9600	Cr2—O3	1.986 (3)
C8—H8B	0.9600	Cr2—O4	2.009 (3)
C8—H8C	0.9600	Cr2—N1	2.021 (4)
C9—O11	1.245 (4)	Cr3—O1	1.900 (3)
C9—O10	1.262 (5)	Cr3—O11	1.964 (2)
C9—C10	1.536 (4)	Cr3—O12	1.976 (3)
C10—H10A	0.9600	Cr3—O5	1.983 (3)
C10—H10B	0.9600	Cr3—O6	2.000 (2)
C10—H10C	0.9600	Cr3—O15	2.032 (3)
C11—O12	1.239 (5)	N1—N2	1.305 (4)
C11—O13	1.264 (5)	N2—N3	1.059 (4)
C11—C12	1.538 (7)	O14—H14D	0.8179
C12—H12A	0.9600	O15—H15A	0.8296
C12—H12B	0.9600	O16—H16F	0.9601
C12—H12C	0.9600		
			
O2—C1—O3	125.7 (4)	H16B—C16—H16C	109.5
O2—C1—C2	118.3 (3)	O1—Cr1—O8	96.06 (10)
O3—C1—C2	116.0 (4)	O1—Cr1—O2	96.40 (13)
C1—C2—H2A	109.5	O8—Cr1—O2	90.44 (12)
C1—C2—H2B	109.5	O1—Cr1—O13	95.49 (13)
H2A—C2—H2B	109.5	O8—Cr1—O13	88.55 (12)
C1—C2—H2C	109.5	O2—Cr1—O13	168.11 (11)
H2A—C2—H2C	109.5	O1—Cr1—O7	95.18 (10)
H2B—C2—H2C	109.5	O8—Cr1—O7	168.75 (11)
O4—C3—O5	126.6 (4)	O2—Cr1—O7	88.29 (12)
O4—C3—C4	114.2 (4)	O13—Cr1—O7	90.40 (13)
O5—C3—C4	119.1 (3)	O1—Cr1—O14	177.65 (16)
C3—C4—H4A	109.5	O8—Cr1—O14	83.99 (10)
C3—C4—H4B	109.5	O2—Cr1—O14	85.95 (13)
H4A—C4—H4B	109.5	O13—Cr1—O14	82.16 (13)
C3—C4—H4C	109.5	O7—Cr1—O14	84.77 (10)
H4A—C4—H4C	109.5	O1—Cr2—O10	94.44 (12)
H4B—C4—H4C	109.5	O1—Cr2—O9	93.78 (11)
O6—C5—O7	126.5 (3)	O10—Cr2—O9	89.29 (11)
O6—C5—C6	117.6 (3)	O1—Cr2—O3	93.94 (11)
O7—C5—C6	115.8 (3)	O10—Cr2—O3	171.55 (13)
C5—C6—H6A	109.5	O9—Cr2—O3	91.26 (11)
C5—C6—H6B	109.5	O1—Cr2—O4	93.63 (11)
H6A—C6—H6B	109.5	O10—Cr2—O4	90.47 (11)
C5—C6—H6C	109.5	O9—Cr2—O4	172.58 (12)
H6A—C6—H6C	109.5	O3—Cr2—O4	87.90 (11)
H6B—C6—H6C	109.5	O1—Cr2—N1	175.94 (14)
O8—C7—O9	126.2 (3)	O10—Cr2—N1	89.56 (13)
O8—C7—C8	119.2 (3)	O9—Cr2—N1	85.55 (12)
O9—C7—C8	114.6 (3)	O3—Cr2—N1	82.08 (13)
C7—C8—H8A	109.5	O4—Cr2—N1	87.03 (12)
C7—C8—H8B	109.5	O1—Cr3—O11	94.65 (10)
H8A—C8—H8B	109.5	O1—Cr3—O12	95.62 (13)
C7—C8—H8C	109.5	O11—Cr3—O12	88.27 (11)
H8A—C8—H8C	109.5	O1—Cr3—O5	95.98 (13)
H8B—C8—H8C	109.5	O11—Cr3—O5	92.11 (11)
O11—C9—O10	125.4 (3)	O12—Cr3—O5	168.32 (11)
O11—C9—C10	118.3 (3)	O1—Cr3—O6	93.59 (10)
O10—C9—C10	116.3 (3)	O11—Cr3—O6	171.59 (12)
C9—C10—H10A	109.5	O12—Cr3—O6	89.24 (11)
C9—C10—H10B	109.5	O5—Cr3—O6	88.72 (11)
H10A—C10—H10B	109.5	O1—Cr3—O15	179.66 (11)
C9—C10—H10C	109.5	O11—Cr3—O15	85.02 (10)
H10A—C10—H10C	109.5	O12—Cr3—O15	84.44 (13)
H10B—C10—H10C	109.5	O5—Cr3—O15	83.97 (12)
O12—C11—O13	126.4 (5)	O6—Cr3—O15	86.74 (10)
O12—C11—C12	117.4 (4)	N2—N1—Cr2	119.4 (3)
O13—C11—C12	116.2 (4)	N3—N2—N1	166.3 (4)
C11—C12—H12A	109.5	Cr1—O1—Cr3	121.05 (16)
C11—C12—H12B	109.5	Cr1—O1—Cr2	119.72 (15)
H12A—C12—H12B	109.5	Cr3—O1—Cr2	119.22 (14)
C11—C12—H12C	109.5	C1—O2—Cr1	130.8 (2)
H12A—C12—H12C	109.5	C1—O3—Cr2	134.2 (3)
H12B—C12—H12C	109.5	C3—O4—Cr2	130.0 (3)
O14—C13—H13A	109.5	C3—O5—Cr3	133.3 (2)
O14—C13—H13B	109.5	C5—O6—Cr3	131.1 (2)
H13A—C13—H13B	109.5	C5—O7—Cr1	132.4 (2)
O14—C13—H13C	109.5	C7—O8—Cr1	133.5 (2)
H13A—C13—H13C	109.5	C7—O9—Cr2	131.88 (17)
H13B—C13—H13C	109.5	C9—O10—Cr2	134.9 (2)
O15—C14—H14A	109.5	C9—O11—Cr3	130.3 (3)
O15—C14—H14B	109.5	C11—O12—Cr3	133.7 (3)
H14A—C14—H14B	109.5	C11—O13—Cr1	130.1 (3)
O15—C14—H14C	109.5	C13—O14—Cr1	123.2 (3)
H14A—C14—H14C	109.5	C13—O14—H14D	112.4
H14B—C14—H14C	109.5	Cr1—O14—H14D	124.1
O16—C16—H16A	109.5	C14—O15—Cr3	126.4 (3)
O16—C16—H16B	109.5	C14—O15—H15A	124.3
H16A—C16—H16B	109.5	Cr3—O15—H15A	98.1
O16—C16—H16C	109.5	C16—O16—H16F	109.2
H16A—C16—H16C	109.5		
			
O1—Cr2—N1—N2	−150.2 (17)	C6—C5—O6—Cr3	176.4 (3)
O10—Cr2—N1—N2	19.8 (3)	O1—Cr3—O6—C5	29.2 (4)
O9—Cr2—N1—N2	−69.5 (3)	O11—Cr3—O6—C5	−139.1 (8)
O3—Cr2—N1—N2	−161.4 (3)	O12—Cr3—O6—C5	−66.3 (4)
O4—Cr2—N1—N2	110.3 (3)	O5—Cr3—O6—C5	125.2 (4)
Cr2—N1—N2—N3	−110 (2)	O15—Cr3—O6—C5	−150.8 (4)
O8—Cr1—O1—Cr3	−136.06 (16)	O6—C5—O7—Cr1	−9.9 (8)
O2—Cr1—O1—Cr3	132.82 (15)	C6—C5—O7—Cr1	171.7 (3)
O13—Cr1—O1—Cr3	−46.94 (15)	O1—Cr1—O7—C5	−10.8 (4)
O7—Cr1—O1—Cr3	43.96 (18)	O8—Cr1—O7—C5	169.4 (7)
O14—Cr1—O1—Cr3	−45 (2)	O2—Cr1—O7—C5	−107.0 (4)
O8—Cr1—O1—Cr2	42.87 (18)	O13—Cr1—O7—C5	84.8 (4)
O2—Cr1—O1—Cr2	−48.26 (15)	O14—Cr1—O7—C5	166.9 (4)
O13—Cr1—O1—Cr2	131.98 (15)	O9—C7—O8—Cr1	−5.3 (7)
O7—Cr1—O1—Cr2	−137.11 (17)	C8—C7—O8—Cr1	177.1 (3)
O14—Cr1—O1—Cr2	134 (2)	O1—Cr1—O8—C7	−13.9 (4)
O11—Cr3—O1—Cr1	128.45 (16)	O2—Cr1—O8—C7	82.6 (4)
O12—Cr3—O1—Cr1	39.74 (16)	O13—Cr1—O8—C7	−109.3 (4)
O5—Cr3—O1—Cr1	−138.94 (15)	O7—Cr1—O8—C7	166.0 (7)
O6—Cr3—O1—Cr1	−49.85 (17)	O14—Cr1—O8—C7	168.5 (4)
O15—Cr3—O1—Cr1	140 (50)	O8—C7—O9—Cr2	−5.1 (6)
O11—Cr3—O1—Cr2	−50.48 (17)	C8—C7—O9—Cr2	172.6 (3)
O12—Cr3—O1—Cr2	−139.20 (14)	O1—Cr2—O9—C7	29.4 (4)
O5—Cr3—O1—Cr2	42.13 (15)	O10—Cr2—O9—C7	123.8 (4)
O6—Cr3—O1—Cr2	131.22 (16)	O3—Cr2—O9—C7	−64.6 (4)
O15—Cr3—O1—Cr2	−39 (26)	O4—Cr2—O9—C7	−148.0 (8)
O10—Cr2—O1—Cr1	−137.26 (15)	N1—Cr2—O9—C7	−146.6 (4)
O9—Cr2—O1—Cr1	−47.68 (16)	O11—C9—O10—Cr2	−15.3 (6)
O3—Cr2—O1—Cr1	43.85 (16)	C10—C9—O10—Cr2	163.1 (2)
O4—Cr2—O1—Cr1	131.99 (15)	O1—Cr2—O10—C9	−5.6 (3)
N1—Cr2—O1—Cr1	32.8 (19)	O9—Cr2—O10—C9	−99.3 (3)
O10—Cr2—O1—Cr3	41.69 (16)	O3—Cr2—O10—C9	166.9 (6)
O9—Cr2—O1—Cr3	131.27 (15)	O4—Cr2—O10—C9	88.1 (3)
O3—Cr2—O1—Cr3	−137.21 (15)	N1—Cr2—O10—C9	175.1 (3)
O4—Cr2—O1—Cr3	−49.07 (16)	O10—C9—O11—Cr3	−1.6 (5)
N1—Cr2—O1—Cr3	−148.3 (17)	C10—C9—O11—Cr3	−180.0 (2)
O3—C1—O2—Cr1	−9.1 (5)	O1—Cr3—O11—C9	32.0 (3)
C2—C1—O2—Cr1	167.7 (2)	O12—Cr3—O11—C9	127.5 (3)
O1—Cr1—O2—C1	30.3 (3)	O5—Cr3—O11—C9	−64.2 (3)
O8—Cr1—O2—C1	−65.8 (3)	O6—Cr3—O11—C9	−159.7 (8)
O13—Cr1—O2—C1	−150.8 (4)	O15—Cr3—O11—C9	−148.0 (3)
O7—Cr1—O2—C1	125.4 (3)	O13—C11—O12—Cr3	−5.7 (5)
O14—Cr1—O2—C1	−149.8 (3)	C12—C11—O12—Cr3	175.46 (19)
O2—C1—O3—Cr2	2.7 (5)	O1—Cr3—O12—C11	−10.1 (3)
C2—C1—O3—Cr2	−174.2 (2)	O11—Cr3—O12—C11	−104.6 (3)
O1—Cr2—O3—C1	−19.3 (3)	O5—Cr3—O12—C11	163.4 (4)
O10—Cr2—O3—C1	168.2 (6)	O6—Cr3—O12—C11	83.5 (3)
O9—Cr2—O3—C1	74.6 (3)	O15—Cr3—O12—C11	170.3 (3)
O4—Cr2—O3—C1	−112.8 (3)	O12—C11—O13—Cr1	−7.8 (5)
N1—Cr2—O3—C1	159.9 (3)	C12—C11—O13—Cr1	171.06 (19)
O5—C3—O4—Cr2	−21.2 (5)	O1—Cr1—O13—C11	31.7 (3)
C4—C3—O4—Cr2	163.1 (2)	O8—Cr1—O13—C11	127.6 (3)
O1—Cr2—O4—C3	39.6 (3)	O2—Cr1—O13—C11	−147.2 (4)
O10—Cr2—O4—C3	−54.9 (3)	O7—Cr1—O13—C11	−63.6 (3)
O9—Cr2—O4—C3	−143.0 (8)	O14—Cr1—O13—C11	−148.2 (3)
O3—Cr2—O4—C3	133.4 (3)	O1—Cr1—O14—C13	−150 (2)
N1—Cr2—O4—C3	−144.4 (3)	O8—Cr1—O14—C13	−58.2 (3)
O4—C3—O5—Cr3	7.1 (5)	O2—Cr1—O14—C13	32.6 (2)
C4—C3—O5—Cr3	−177.4 (2)	O13—Cr1—O14—C13	−147.6 (3)
O1—Cr3—O5—C3	−17.4 (3)	O7—Cr1—O14—C13	121.3 (3)
O11—Cr3—O5—C3	77.5 (3)	O1—Cr3—O15—C14	139 (26)
O12—Cr3—O5—C3	169.1 (4)	O11—Cr3—O15—C14	150.3 (4)
O6—Cr3—O5—C3	−110.9 (3)	O12—Cr3—O15—C14	−121.0 (4)
O15—Cr3—O5—C3	162.3 (3)	O5—Cr3—O15—C14	57.7 (3)
O7—C5—O6—Cr3	−2.0 (7)	O6—Cr3—O15—C14	−31.4 (4)

### Hydrogen-bond geometry (Å, °)

**Table:**

*D*—H···*A*	*D*—H	H···*A*	*D*···*A*	*D*—H···*A*
O16—H16F···N1^i^	0.96	1.85	2.734 (4)	151.
O16—H16F···O3^i^	0.96	2.36	2.972 (3)	122.
O16—H16F···O9^i^	0.96	2.54	3.254 (5)	132.
O16—H16F···N2^i^	0.96	2.63	3.370 (5)	134.
O15—H15A···N1^ii^	0.83	1.91	2.621 (5)	144.
O15—H15A···N2^ii^	0.83	2.58	3.332 (5)	152.
O14—H14D···O16	0.82	2.03	2.602 (5)	126.

Symmetry codes: (i) *x*, −*y*+1, *z*+1/2; (ii) *x*, −*y*, *z*+1/2.
